# Breaking Down the Stigma: A Review of the Literature on the Relationships between Autism Spectrum Disorder and Criminal Behavior

**DOI:** 10.3390/brainsci14100984

**Published:** 2024-09-28

**Authors:** Liliana Dell’Osso, Benedetta Nardi, Martina Calvaruso, Lucrezia Castellani, Cristiana Pronestì, Ivan Mirko Cremone, Stefano Pini, Barbara Carpita

**Affiliations:** Department of Clinical and Experimental Medicine, Section of Psychiatry, University of Pisa, 67 Via Roma, 56126 Pisa, Italy; liliana.dellosso@unipi.it (L.D.); benedetta.nardi@live.it (B.N.); m.calvaruso@gmail.com (M.C.); castellanilucrezia91@gmail.com (L.C.); c.pronesti@studenti.unipi.it (C.P.); ivan.cremone@gmail.com (I.M.C.); stefano.pini@unipi.it (S.P.)

**Keywords:** autism spectrum disorder, autism, criminality, offenders, criminal justice system

## Abstract

**Background:** In recent years, there has been growing interest in the evaluation of autism spectrum disorder (ASD) and autistic traits in prison populations and offenders. Due to misleading headlines and highly publicized criminal cases, the belief that autistic individuals are more prone to commit crimes has spread among the general population, also leading to increasing research on this matter. **Aims**: In this context, this narrative review aimed to analyze the available scientific literature on the bi-directional link between ASD and criminal behaviors and to assess the key characteristics of eventual ASD offenders, including sociodemographic data, comorbidities, crime-related features, and interactions with the criminal justice system. **Results:** Our review highlighted that the available studies lack methodological rigor and present controversial results. Overall, the current state of research does not support any definitive correlation between ASD or autistic traits and the predisposition to engage in criminal conduct. Further studies are needed to confirm or reject this hypothesis.

## 1. Introduction

Autism spectrum disorder (ASD) is a common neurodevelopmental condition, estimated to affect more than 1% of the general population [1]. It is characterized by a pervasive and sustained deficit in social communication and social interaction, restrictive and repetitive patterns of behavior, interests, or activities, and hyper- or hypo-reactivity to sensory stimuli [2]. However, ASD is an extremely heterogeneous disorder, with individuals presenting different grades of intellectual disability or language impairment [3]. Today, it is recognized that ASD varies along a continuum of manifestations, ranging from the more common ones to those more atypical or attenuated. Indeed, the latest edition of the Diagnostic and Statistical Manual for Mental Disorders (DSM-V-TR) merged most of the previous diagnoses in the concept of spectrum (i.e., Autistic Disorder, Asperger’s Syndrome, Pervasive Developmental Disorder) [2]. Moreover, autistic traits have been documented in the general population, with a higher prevalence among psychiatric patients. These traits have been suggested to represent a major risk factor for the development and worsening of many mental disorders as well as for suicidal ideation [4,5,6,7,8,9].

Recently, there has been a growing interest in the evaluation of the link between ASD and autistic traits and criminal behaviors [10], with more than 350 papers being published in the past 5 years, according to the popular PubMed database. The belief that autistic subjects may be more prone to commit a crime is spreading among the general population, due to misleading headlines and highly publicized criminal cases [11,12,13]. Moreover, the scientific community still debates the hypothesis that some autistic peculiarities could influence current/future offending behaviors [10,14,15,16]. Some authors claim that because autistic individuals typically follow rules to the letter [16], they are less likely to offend. Others argue that autistic social inexperience, irregularities, sensory overload, and unique interests could contribute to the likelihood of offending behavior [13]. More recently, it was even hypothesized that ASD individuals may have an increased vulnerability to engaging in extremist behaviors when exposed to extremist online material, due to their repetitive interests and compulsive collecting/pursuit [17]. Other hypotheses involve deficits in the *Theory of Mind*, typically present in ASD patients, which may play a role in their altered ability to identify dodges or suspicious behaviors, thereby creating to a vulnerable substrate for both criminal activity and victimization [18].

On the other hand, the available scientific literature regarding the supposed over-representation of individuals with ASD in the criminal justice system (CJS) is still limited and often contradictory [10,14,15,16]. The consequence of the fragmented literature is that the prevalence rate of ASD in offending groups varies greatly depending on the study and the methods of evaluation, ranging from 0.2 to 62.8% [19].

To date, several authors have attempted to draw up reports on the prevalence of crime in ASD and vice versa, but the results continue to be ambiguous. Im et al. [20], in their review concerning violence and ASD, reported that there is no concrete evidence to support an increased violence risk in ASD people compared to the general population. However, the author confirmed the presence of some specific risk factors—not related to ASD—such as younger age, obsessive conduct, other psychiatric comorbidities, emotional regulation, and social cognition deficits that may increase the risk of violence in this group of patients. In contrast, another overview on ASD in adult and juvenile offenders carried out by Rutter found ASD in 2–18% of the forensic groups examined, which is significantly higher than the average prevalence in the general population [21]. Conversely, the prevalence of delinquency in the ASD population was estimated to range from 5 to 26%, which is lower than in the general population [21]. The considerable variability in the prevalence of ASD in both juvenile and adult offenders is likely attributable to the sociocultural context, the use of different diagnostic tools, the variety of the samples examined, the prevalence of concurrent psychiatric problems, and different types of criminal activity.

In 2014, the systematic review by King and Murphy [15] already highlighted a worrying gap in the studies. The authors aimed to assess the prevalence of ASD in CJS and the prevalence of offending behaviors in ASD, as well as the common types of offenses in ASD patients, the comorbidities, and the main characteristics of this population. Although there was an increasing body of literature on these topics, the results were inconclusive due to the wide methodological biases. However, the authors concluded that ASD patients did not seem over-represented in the CJS. More recently, in 2022, Collins et al. [19] aimed to update the previous work of King and Murphy [15]. The authors included 47 articles (20 more than King and Murphy’s review) published from 2013 to 2021, confirming the limited quality of existing research (poor generalizability of the findings, unrepresentative samples, lack of matched comparison sample, reliance on retrospective data collection, lack of standardized instruments). Interestingly, the authors still failed to demonstrate a higher rate of ASD in the CJS.

Moreover, two additional unresolved issues contribute to the ongoing debate: the first is the lack of specific evaluation of the risk of violence for ASD patients at each stage of their transition to the CJS [22], and the second is the inappropriate perception of ASD behavior by the actors involved in the CJS, including police officers, lawyers, and judges [23,24]. A specific evaluation of autistic patients’ specific needs should be advisable, although this confounding scenario highlights how autistic people often do not receive adequate and necessary support during their contact with the CJS.

In this context, our narrative review aimed to analyze the prevalence of ASD and autistic traits among offenders and to assess the key characteristics of this population, including sociodemographic and clinical data, crime-related factors, and interactions with the CJS.

## 2. Materials and Methods

A literature search was conducted using the electronic databases PubMed, Scopus, and Web of Science to identify all potentially eligible records. The following search terms were used, without any filters, restrictions, or limits: ((Autism Spectrum Disorder) OR (Autis*) OR (Autistic traits)) AND ((Crime) OR (Offender) OR (Prisoner) OR (Violence) OR (Criminal)). A manual search was also performed for additional references.

All publications underwent a preliminary screening based on the abstract to assess the consistency of the topic. Full texts were subsequently studied to verify if they met the inclusion criteria:

- in terms of type of article, original research, editorials, and case reports were accepted;
- studies conducted in humans;
- full text available in English;
- studies using psychiatric clinical evaluation or psychometric tests.

Exclusion criteria:

- reviews or meta-analyses;
- articles unavailable in English;
- studies involving neuroimaging procedures.

Articles regarding the prevalence of ASD and autistic traits among offenders, such as the prevalence of offenders among ASD populations, were considered a priority. Studies including original epidemiological and clinical data, criminogenic and criminodynamic factors, together with formal interactions between ASD patients and CJS, were specifically taken into account. Papers lacking crucial data were excluded, while selection based on the sample size, age, or gender was not performed, in line with the purpose of the review.

Each reviewer read the articles independently, and the decision to include them in the review was made as a group. Disagreements between authors were resolved by discussion, although their grade of agreement was generally good.

The selected articles were presented according to the type of criminal act described in the studies, considering the subgroups of general offenders, mass shooters, sex offenders, and cybercriminals. In each subparagraph, the papers followed a chronological order from the oldest to the most recent.

## 3. Results

### 3.1. Offending Behaviors

Most of the current literature groups many criminal acts in the generic term of “offending behaviors”, which includes violence and crimes against persons, property crimes, theft, arson, and others.

One of the first studies assessing the link between crime scene behavior and personality characteristics dates back to 2006 and was carried out by Wahlund and Kristiansson [25]. In their research, the authors performed a retrospective study on 35 male offenders, aged 15 to 71, who were found guilty of manslaughter or homicide and were submitted for forensic psychiatric evaluation in Sweden between 1996 and 2001. All participants were retrospectively diagnosed either with ASD (*n* = 8) or with Antisocial Personality Disorder (APD) (N = 27), according to DSM-IV. Compared to the APD group, offenders belonging to the ASD group were less intoxicated at the time of the offense (56% vs. 90%) and had less history of physical abuse (25% vs. 41%). Moreover, compared to the APD group, ASD offenders were reported to be less likely to use knives or firearms (11% vs. 71%), instead preferring such as poisoning, strangulation, and direct force (80% vs. 28%). The authors, therefore, suggested that personality traits and ASD may have an impact on differences in psychosocial functioning and crime scene features [25].

Allen et al. [11] focused primarily on offenders with Asperger syndrome (AS) and identified 16 people (mean age: 34.8) who had engaged in criminal activity that had or might have brought them into contact with the CJS. Results highlighted that all participants had behavioral issues, particularly 11 (69%) were destructive, 13 (88%) were verbally and 12 (75%) physically aggressive, 11 (69%) exhibited inappropriate sexual behavior, 6 (38%) were over-active, and 6 (38%) used illicit drugs. When evaluating concurrent psychiatric disorders, the most prevalent was schizophrenia (25%), followed by Attention Deficit Hyperactivity Disorder (ADHD) (18.75%), depression (12.5%), anxiety disorders (6.25%), and personality disorders (6.25%). Moreover, the authors highlighted several predisposing factors for offending, including the lack of concern and awareness for the outcome, social naivety, impulsivity, misinterpretation of rules, and overriding obsessions. Furthermore, precipitating factors were identified in social rejection, bullying, sexual rejection, family conflict, deterioration in mental health, change of domicile, and bereavement. Participants in this study had committed an average of three different types of offenses, with the mean age of their first offense being 25.8 years and the type of offense varying from violent conduct (81%), threatening behavior (75%), property destruction (50%), drug offenses and theft (25%), sexual offending (19%), and fraud, motoring offenses, and murder (6%).

Similarly, Newman et al. [26] carried out a review analyzing 37 cases of offenders with AS and evaluated the psychiatric comorbidities reported. Results showed that at the time of perpetrating the crime, 11 subjects (29.7%) had a definite comorbid psychiatric disorder, whereas 20 (54%) had a probable comorbid psychiatric disorder, and in only 6 cases (16.2%), there was no definite proof of a coexisting psychiatric condition. In the group with a definite psychiatric disorder, four offenders manifested bizarre and antisocial acts, one had a body dysmorphic disorder, one had ADHD, and three had mood disorders. In the group with probable psychiatric disorders, one offender had a history of psychiatric admission, one had probable obsessive-compulsive disorder (OCD) and personality disorder, one was diagnosed with a conduct disorder, one with obsessional neurosis, four had a history of fire-setting, and three were admitted to a forensic hospital. Upon those results, the authors suggested that when people with AS commit a crime, the explanation may have as much to do with the AS diagnosis as it has with other elements, such as concomitant mental disorders, that may contribute to the crime’s prevalence in the general community. Indeed, results showed that most AS cases who committed violent crimes also had co-occurring psychiatric illnesses that, as seen in the general population, may increase the risk of engaging in criminal behaviors.

In support of those results, Lundstrom et al. [27] engaged in a longitudinal study on the long-term effects of ASD, ADHD, OCD, and tic disorders on criminality by using six registers of all children and adolescents receiving services for mental health in Stockholm, Sweden. The authors ultimately identified 3391 children, of whom 1366 had an ADHD diagnosis, 954 had an ASD diagnosis, 214 had tic disorders, and 857 had an OCD diagnosis. Diagnoses were made by psychiatrists, licensed psychologists, and social workers and validated by Autism, Tic, ADHD, and other Comorbidities Inventory (A-TAC). When compared to a sample of randomly chosen population controls (N = 33,910), matched for age, gender, and residential location, ASD or OCD groups were not associated with an increased risk of committing violent crimes, while ADHD or tic disorders were. Furthermore, the familial context (i.e., family and marital conflicts) could play a role in the link between ADHD and criminality, as highlighted by the slight but noticeable higher risk for committing violent crimes reported among siblings of ADHD subjects. On the other hand, adult mental illness, including substance addiction, did not appear to be a risk factor for criminality.

In another study, Helverschou et al. [28] tried to identify offending profiles of individuals with ASD. For their purpose, they described 48 subjects (M = 41; F = 7) diagnosed with ASD according to the ICD-10. Interestingly, in most of the cases, the ASD diagnosis was made late in life (mean age: 25.3 years), and in 22 of the 48 cases, it was made for the first time by the forensic experts who were assessing the offenders. Even in this case, comorbid diagnoses were frequent (83%) and included personality disorders, psychosis, and several others; in addition, 16 offenders (33%) had an intellectual disability, 18 reported alcohol or other substance use (38%), and 10 (21%) were under the influence of drugs during the crime. The most frequent type of crime was violent (44%), followed by sex crimes (25%). Interestingly, most ASD participants in this study had a close relationship with their victims, exhibited no signs of substance misuse, and were eager to confess to the alleged crime—unlike most other criminals. Moreover, based on the forensic reports, most of the illegal activities (n = 42; 88%) were regarded as being deliberately planned, and frequent reasons were reported as idiosyncratic belief systems, obsessions, and/or particular interests. The authors concluded that there was no conclusive link between criminal acts and ASD characteristics, although the idiosyncratic belief system and obsessions seemed to be connected to the crime’s motivation.

Different conclusions were reported in the study by Loureiro et al., published in 2018 [29], which investigated the association between autism, criminality, and psychopathy. In this study, several self-reporting questionnaires were administered to 101 high-security prison inmates and a control group. The prevalence of autistic traits was specifically measured by using the Autism Spectrum Quotient (AQ), while psychopathy was measured by the Triarchic Psychopathy Measure (TriPM). The Brief Symptom Inventory (BSE) and the Adult ADHD Self-Reported Scale (ASRS) were also employed, to evaluate additional clinical features. The results revealed that prisoners had more autistic traits (*p* = 0.002) than the control group, mainly due to higher scores in the domains of communication and imagination. Conversely, no correlations were reported between autistic and psychopathy traits. The authors concluded that autistic traits are an independent risk factor for imprisonment, although no correlation with psychopathy was detected.

Similarly to adults, various studies, focusing on juvenile offenders, did not find a link between ASD and the risk of committing a crime. In detail, Slaughter et al. [30], in 2019, matched a group of 143 justice-involved youth (JJY) with ASD to a sample of JJY without ASD (N = 286). Results revealed that JJYs with ASD were considerably less likely to commit property offenses, suggesting that the presence of executive functioning deficits may affect the capacity to plan, organize, and carry out crimes like vandalism, robbery, and burglary covertly. The hypothesis is that subjects with ASD could be less likely to commit crimes that involve premeditation and more likely to participate in illicit behaviors that result from impulsive encounters [30,31,32]. Furthermore, results showed overall no significant differences in the type of school discipline incidents between the two groups. Regarding crimes against people, there were no observed differences between the two groups. Besides, there was no significant difference between the two groups regarding individual school discipline types (threats, fighting, brawls, and infractions of school rules). Furthermore, JJY who had a history of fighting in school were more likely to recidivate regardless of their special education classification. Ultimately, the authors concluded that, when compared to youngsters without ASD, ASD subjects are not more likely to commit crimes.

In the same year, Hofvander et al. [33] investigated the prevalence of ASD in 269 young violent offenders from the national cohort of the Development of Aggressive Antisocial Behavior Study (DAABS). Twenty-six of these (10% of the sample) were diagnosed with ASD according to DSM-IV criteria and were compared with the remaining sample (243 non-ASD offenders) for externalizing behaviors and criminal history. The two groups were similar in median age (21.6 vs. 22.2, *p* = 0.09). Participants were assessed by the Structured Clinical Interview for DSM (SCID), the Asperger Syndrome Diagnostic Interview (ASDI), the Autism-Tics ADHD and Other Comorbidities Inventory (A-TAC), and the Psychopathy Checklist—Revised (PCL-R). Interestingly, the total PCL-R scores did not differ significantly, although ASD offenders scored higher in the Affect facet domain. Overall, the authors found a higher prevalence of externalizing and antisocial behaviors in the whole sample, with few differences between the two groups. In detail, ASD offenders were more likely to report convictions related to sex crimes against child victims (15.4% vs. 4.1%; *p* < 0.05), while the non-ASD group to drug-related crimes (4.6% vs. 3.1%; *p* < 0.05). Moreover, non-ASD offenders had more often a substance use disorder in comorbidity and a higher number of convictions. Foster home placement was also over-reported in the ASD group. Notably, there was no difference in the total number of crimes between the young ASD and non-ASD offenders, confirming previous results.

Recently, van Buitenen et al. [34] collected data from 394 male offenders with an ASD diagnosis from penitentiary psychiatric centers in the Netherlands to evaluate the prevalence rates of comorbid disorders. The results highlighted a comorbid mental disorder in 78.9% of the sample, with the most frequent being substance use disorder (39.8%), other neurodevelopmental disorders (24.1%), and schizophrenia spectrum disorders (31.7%). Since these disorders were previously linked to violent behaviors, the authors suggested that there is no direct connection between ASD and the perpetration of violent crimes, while comorbidity, influenceability, and a detrimental social network are better indicators of violence in this context.

Yu et al. [35] used the large epidemiological dataset of the South Carolina Autism and Developmental Disabilities Monitoring (SC ADDM) Network to conduct a subset of the Carolina Autism Transition Study (CATS), a larger project involving multiple state agencies. The study analyzed a sample of 4850 participants, aged 2–8 years, over a period of 15 years (2000–2015). Data regarding the CJS involvement were collected from the Department of Juvenile Justice (DJJ) and the SC Law Enforcement Division (SLED) for individuals under and over 18 years old, respectively. Subjects were divided into ASD (N = 606; 81% male) and intellectual disability (ID; N = 1271; 64% male) groups, while the population control group (PC; N = 2973; 81% male) was selected using the birth certificate records. The percentage of subjects involved in the juvenile CJS did not differ significantly between the three groups (3% of ASD; 7% of ID; 5% of PC). Conversely, the ASD group (*p* = 0.0122) and the ID group (*p* = 0.0003) were significantly less involved in the adult CJS, compared to the PC group. In both juvenile and adult CJSs, the ASD group did not differ from the PC group in the total number of charges and incidents, age of the first contact with CJS, and charge categories. Nevertheless, young and adult ASD individuals committed a few more crimes against persons, offenses against public order, and crimes against property. Moreover, young ASD subjects were less charged with sex crimes if compared with the ID or PC group (6% vs. 13% vs. 7%), although this difference was not statistically significant. Lastly, recidivism was similar among the groups, although ASD subjects showed lower rates in comparison to ID or PC. The authors concluded that ASD individuals were not over-represented in the CJS in comparison to non-ASD, being similarly involved in the juvenile CJS and less in the adult one.

A New Zealand national birth cohort study [36] compared young adults between 17 and 25 years of age, with (N = 1197) and without (N = 146.863) ASD, in their interactions with the CJS. After adjusting for socio-demographic factors, young adults with ASD had lower rates of being investigated by the police, accused in court, and found guilty. However, those accused of a crime were much more likely to be charged with major offenses, violent offenses, offenses against people, and offenses against property.

In 2022, Blackmore et al. [37] conducted a retrospective review of 1570 medical records (1140 males and 428 females aged 17–75 years) assessed for ASD from April 2003 to February 2020. ASD diagnosis resulted in 1130 subjects (72% of the sample) according to the International Statistical Classification of Diseases, Injuries and Causes of Death (ICD) criteria. The Autism Diagnostic Interview—Revised (ADI-R) and/or the Autism Diagnostic Observational Schedule (ADOS-G or ADOS-2) were also employed to confirm the results. Compared to the non-ASD patients (N = 440), the ASD group had a lower prevalence of any CJS contact (23% vs. 32%; *p* < 0.01). In detail, violent offenses (10% vs. 14%), theft/burglary (5% vs. 9%), and substance offenses (3% vs. 6%) were less likely committed by the ASD group. Conversely, no significant differences were found in sexual crimes, criminal damage, arson, stalking/harassment, and other offenses between the two groups. Moreover, ASD had more co-currying ADHD (20% vs. 11%) and anxiety disorders (51% vs. 43%) in comparison to the non-ASD group, and significantly fewer personality disorders (3% vs. 5%) or psychotic disorders (3% vs. 6%). Interestingly, male sex (OR = 3.5, *p* < 0.001) or having ADHD (OR = 1.8, *p* < 0.001) and/or a psychotic disorder (OR = 2.2, *p* = 0.021) were found to be risk factors for the involvement in CJS of ASD patients. The authors suggested that ASD and non-ASD patients did not differ significantly in CJS involvement and that modifying treatable risk factors could contribute to reducing ASD contact with the CJS.

Another study conducted by Hofvander et al. [38], aimed to investigate the patterns of comorbidity and criminality in a sample of 831 ASD offenders, who had received a psychiatric forensic investigation between 2001 and 2018 in Sweden. The sample included 708 men and 123 women (mean age 29.9 years) with ASD diagnosis, according to the DSM-IV criteria. Results revealed that 66.7% of them had at least one other concomitant psychiatric diagnosis: substance use disorder (26.0%) was the most frequent, followed by schizophrenia (16.1%), ID (15.8%), and ADHD (15.4%). Among the crime categories, violent crimes (75.5%) were the more common, followed by sexual offenses (16.1%), vandalism (13.1%), theft (10.3%), drug-related crimes (7.3%), and others (36.8%). The authors finally identified three general clinical profiles of ASD offenders: ASD with mainly neurodevelopmental problems, ASD with different externalizing problems, and ASD with complex personality-related disorders. They suggested that all parts of the CJS, including treatment and psychosocial interventions, may need to be prepared and adapted to at least these three ASD subgroups.

A summary of the studies described is included in Table 1.

### 3.2. Mass Shooting

Mass shootings usually attract significant media attention due to the unpredictability of these events and the large number of people involved. In one of the most comprehensive papers on mass shootings and the potential presence of ASD features in the perpetrators, Allely et al. [39] collected data from 73 mass shooting events that occurred in the USA from 1982 to 2015. The authors hypothesized the presence of significant ASD characteristics in 6 out of the 75 mass shooters, based on suggestions from other databases and information provided by family and friends. Furthermore, the authors described ASD features in an additional 16 mass shooters. However, based on those results, the authors hinted that individuals with ASD are not more likely to be mass shooters or commit crimes, but having ASD, in some circumstances, may exacerbate other problems and make them more difficult to deal with. Moreover, in most cases, there were concomitant psychiatric diagnoses and additional environmental risk factors, including experiencing abuse in childhood.

A summary of the study described above is included in Table 2.

### 3.3. Sex-Offenders

Several studies have specifically focused on sex-related crimes, a subgroup of crimes against persons that differ significantly from the others due to the wide gender gap between offenders (mostly males) and victims (mostly females).

One of the first studies investigating autistic traits in sex offenders dates back to 2009 [40]. The authors recruited 175 juvenile males suspected of being sex offenders (mean age: 14.9 ± 1.4), 114 subjects diagnosed with ASD (mean age: 14.2 ± 1.9) and 500 matched healthy controls (mean age: 14.0 ± 1.4). All participants were assessed with the Children’s Social Behavior Questionnaire (CSBQ) and were compared across specific subgroups of sexual offenders, including solo peer offenders, group offenders, and child molesters. The results highlighted higher levels of CSBQ scores in the juvenile sex offenders group compared to the control group, although these levels were still significantly lower compared to the ASD subjects. Moreover, CSBQ scores were higher in child molesters and solo offenders, in comparison to group offenders. These findings underscore the necessity to consider a specific diagnostic assessment and targeted interventions in this peculiar type of juvenile suspected offender.

Years later, Ewoud Baarsma et al. [41] enrolled 44 juvenile sex offenders (mean age: 24.7 ± 1.5) who participated in the previous study and evaluated the stability of their autistic symptoms eight years after they committed the offenses as well as their actual sexual behavior and development. Those parameters were then compared to those of a control group consisting of 52 young males (mean age: 24.3 ± 0.7) selected from a larger representative juvenile sample. Sexuality and sexual development were assessed retrospectively using the Sex under the Age of 25-II questionnaire (SOJ25II), and ASD symptoms, after the first evaluation eight years prior with the CSBQ, were re-assessed by means of the Adult Social Behaviour Questionnaire (ASBQ). Results showed no differences in reaching milestones of sexual development, as well as stability in the autistic symptoms over time. Moreover, no relationship emerged between sexuality and either sexual development or ASD symptoms. The authors suggested that to reduce recidivism among juvenile sexual offenders, it may be important to initiate early treatment to improve social difficulties and offer education and guidance in the area of sexuality.

Payne et al. [42] conducted a semi-structured interview with nine male autistic sexual offenders (mean age: 29.56 ± 8.68) recruited from four prisons and two probation services across England and Wales. The questions focused on the motivation for sexual offending as described by autistic sexual offenders themselves, with the aim of developing interventions to reduce recidivism in this type of offender. The crimes committed included downloading and possessing indecent images, sexual assault, indecent assault, taking and distributing indecent images, causing or inciting a child to engage in sexual activity, and arranging and facilitating a child sex offense. Five main themes, often occurring simultaneously, were reported by autistic sexual offenders as motivations for engaging in their crimes: *social difficulties*, like feeling different from others and loneliness; *misunderstanding*, including being unaware of the consequences or the seriousness of the behavior, a lack of perspective/weak central coherence, misconceptions about what is available online, misunderstanding the rules, and reasoning differences; *sex and relationship deficits*, such as weak sexual experience, a desire for sexual experience, misunderstanding consent, lack of appropriate relationships, and having transgressive relationships; *inadequate impulse control*, including getting carried away, immaturity, and excuses; *disequilibrium* following significant life events, instability, lack of professional support, lack of familial support, and use of substances. Notably, it became clear that the first two themes reflect distinct ASD-related difficulties. Based on the reported reasons for offending and the lack of sexual knowledge and awareness of autistic sexual offenders, this research highlights the need to develop specific sex and relationship education interventions for these patients.

Lastly, Rutten et al. [43] investigated whether different neurodevelopment disorders, such as ASD, ADHD, or both, were correlated with different types of alleged index offenses. For this purpose, the authors recruited a sample of 188 male adolescents, 69 of whom had a diagnosis of ASD, 90 with ADHD, and 29 with both disorders. The subjects were assessed with a 76-item checklist. The results showed that the rate of sex offenses was significantly higher among male adolescents with ASD than those with ADHD or with comorbid ASD and ADHD, while no differences were found in the violent offending rates. The authors suggested the necessity of specific interventions in adolescent offenders with different neurodevelopment disorders, focusing on emotion recognition and its association with social interaction for patients with ASD and on family and relevant educational areas for patients with ADHD.

A summary of the study described above is included in Table 3.

### 3.4. Cybercrime

Cybercrime is a growing area of criminality, closely related to the widespread proliferation of digital technologies and social media. Hackers, virus writers, cyberbullies, and identity thieves are more and more frequent worldwide [44].

In 2014, Seigfried-Spellar et al. [45] assessed the possible relationship between cyber-deviancy and autistic traits in a sample of 296 university students. The participants completed a demographics questionnaire, the AQ, and a survey measuring self-reported computer deviant behaviors named Computer Crime Index—Revised (CCI-R). The results showed that 60% of the students reported some form of computer deviant behavior, but only 0.01% obtained an AQ score clinically significant for AS. Interestingly, both the AQ and subscale scores reported by non-hackers were higher than those of the hackers’ groups. Moreover, cyberbullies obtained higher scores in the total AQ and in poor communication skills compared to non-cyberbullies. Identity thieves and virus writers reported higher scores in the total AQ and presented poorer scores in imagination, social skills, and communication compared to their non-deviant counterparts. Furthermore, results showed that participants who self-reported engaging in all four types of cyber-deviancies had higher scores in the total AQ as well as more problems with imagination, social skills, and communication. The authors therefore suggested the greater prevalence of autistic-like traits in individuals who committed computer crimes compared to those who did not.

The study by Payne et al. [46], published in 2019, was the first to empirically explore the potential relationships between cyber-dependent crime and autistic-like traits, autism, perceived interpersonal support, and explicit social cognition. The sample included 290 internet users without previous contact with the CJS (mean age of 24.24 ± 9.25), 23 of whom self-reported being autistic. All participants were assessed with the brief assessments of non-verbal IQ scored with the Ravens Advanced Progressive Matrices Set I (RAPM), the AQ, the informal test of social know-how (IToSK), the Interpersonal Support Evaluation List-12 (ISEL-12) for perceived interpersonal support, the Basic and Advanced Digital Skills (BADS), and the Cyber-Dependent Crime Questionnaire (CDCQ), designed specifically for this study. The 23 participants who self-reported as autistic, consistently showed higher levels of autistic-like traits than the others, as measured by the AQ. Internet users who had carried out one or more cyber-dependent crimes presented greater basic and advanced digital skills and higher AQ scores and were less likely to report themselves as autistic. Moreover, results showed an increased risk of committing cyber-dependent crimes in individuals with higher autistic-like traits, although a diagnosis of ASD was associated with a decreased risk. Ultimately, having advanced digital skills mediated 40% of the association between cyber-dependent crime and autistic-like traits. The authors suggested that some members of the autistic community may be ideally suited to cybersecurity employment.

The same authors, in 2020 [47] investigated the self-reported motivations for engaging in or declining to engage in cyber-offending and their relation to the level of autistic traits in the participants. The study recruited 182 subjects, of whom 175 were non-offenders and 7 were cyber-dependent offenders (CDO). Of the 175 non-offenders, 29 (mean age: 28.59 ± 9.16) reported being approached to commit a cyber-dependent offense but had declined and were, therefore, indicated as cyber-dependent decliners (CDD). The offenders committed computer misuse offenses like creating, supplying, modifying, obtaining computer materials, or having unauthorized access to these. Every participant completed the RAPM, the AQ-50 for the assessment of autistic traits, the BADS, and questions regarding the motivations for engaging in or declining to engage in cyber-depending crimes. The CDD group presented a higher IQ than the CDO group. The CDO group also reported seven main motivations for engaging in cyber-dependent offending: lack of understanding, entertainment, peer influence, experience and career, anonymity and risk perception, life events, and morals. Conversely, the CDD group expressed seven different main motivations for declining to commit a cyber-dependent offense, such as moral principles, perception of risk, fear of consequences, not wanting to, wanting to adhere to the law, behavior being too complicated, and the rewards being too low. Notably, only one participant of the CDO group reported an ASD diagnosis. Moreover, the mean AQ-50 score for the CDO group was lower than that for the CDD group and lower than the cut-off of 26 proposed for ASD diagnosis [48]. Interestingly, in the CDD group, instrumental themes such as cyber-offending being too risky or not profitable enough were associated with an AQ score averaging above the cut-off of 26, while themes regarding morals/being law-abiding were associated with an average AQ score below. These findings may suggest that autistic traits relate to reasons for declining to commit cyber-dependent offenses and may provide a starting point for the development of targeted programs to reduce cyber-dependent crimes.

Lastly, an online survey conducted with 302 subjects, 25 of whom had a diagnosis of ASD, investigated the relationship between autistic traits, ASD, and cybercrime [49]. All participants completed the AQ-12 for the assessment of autistic traits, a 10-item basic digital skills questionnaire, a 10-item advanced digital skills questionnaire, the social subscale of the Adult Theory of Mind—Quick test (A-ToM-Q), Part 1 of the Advanced Vocabulary Test I–V-4 (AVT) to measure the level of verbal comprehension, the CDCQ, and the CCI-R. As expected, individuals with a diagnosis of autism scored significantly higher in the AQ-12 than individuals without. Regarding the CCI-R results, ASD subjects reported more engagement in criminal activity, with younger age being predictive of cybercriminal activity. However, gender, basic/advanced digital skills, A-ToM-Q, AQ-12, and AVT scores did not present significant correlations with the endorsement of cybercrime, except for the engagement in unauthorized network monitoring that was significantly less reported in individuals with higher autistic traits. On the other hand, when measuring cybercriminal activity using the CDCQ, verbal comprehension and advanced digital skills were associated with more cyber-dependent crime. Therefore, the authors suggested that additional factors, such as understanding of cybercrime, willingness to disclose criminal activity, and specific autistic characteristics, may affect the complex relationship between autism, autistic traits, and cybercrime.

A brief summary of the study described above is included in Table 4.

## 4. Discussion

In recent years, dramatized headlines and news provided by the media have often led to the spreading belief, in the general population, that subjects on the autism spectrum are more likely to engage in some kind of criminal behavior [11,36]. Such press attention seems particularly troublesome because it makes unfounded claims about the connection between criminal behavior and ASD that could lead to unjustified and unfavorable public reactions, such as stigmatization of those on the spectrum [50]. Furthermore, there is minimal proof that the perpetrators have a formal ASD diagnosis, and when they do, they are extreme cases with extremely low frequencies of incidence in the general community [50,51]. Indeed, extensive revisions of research addressing the relationship between criminal conduct and ASD have not identified a direct correlation between violent criminal activity and the autism spectrum [52,53]. Moreover, in the past decades, research on ASD and criminal activity has recognized two primary categories of investigation, such as the prevalence of ASD and autistic traits in offenders and the prevalence of CJS interactions with subjects on the spectrum [15,54]. Prior studies suggested that adults with ASD may be overrepresented in certain facets of the CJS, with some data suggesting that people with autism are more likely than the general population to interact with law enforcement [55,56]. However, the rates of subjects with ASD or autistic-like features in the CJS have changed significantly over time, perhaps as a result of modernization in the methodological techniques, with more recent research suggesting that there is no significant difference in the likelihood of autistic individuals and non-autistic individuals coming into contact with the CJS [16,31,57,58,59]. Nevertheless, there is still a strong ongoing disagreement regarding the relative over- or underrepresentation of autistic individuals in the CJS [14,15,16]. Crucially, many authors reported that people with ASD are more likely to become victims than to commit crimes or act violently. Some studies highlighted how people with developmental disabilities are four to ten times more likely to become victims of crimes [60], ten times more likely to experience sexual assault, and more than twelve times more likely to become victims of robbery [61]. These data are of particular interest considering that the broader category of psychiatric patients, despite the stigma, is reported to show a higher risk of being victims than offenders, while other risk factors such as substance use and socio-environmental features seem to play, as in the general population, a crucial role in criminal behavior [62]. In this framework, it is possible that ASD subjects would be, with respect to other clinical populations, even less prone to become offenders than victims.

The assessment of the ASD prevalence among offenders revealed several criminogenic factors that are also present in non-ASD populations of offenders [19], while ASD offenders have a higher prevalence of psychiatric comorbidities and other potential environmental risk factors [63]. On the other hand, the motivation to offend seems to have some peculiar characteristics in ASD offenders, which differ from the non-ASD offending population [19,28,46,64].

Interestingly, along with prevalence studies, many authors have investigated how perceived behavioral characteristics linked to ASD may influence and predispose individuals to criminal behavior, with some claiming that because autistic individuals typically follow rules to the letter, they are less likely to offend [15] and others arguing that social inexperience, irregularities, sensory overload, and unique hobbies could be contributing factors to the likelihood of offending behavior [13].

In assessing the risk of ASD subjects engaging in illegal conduct, known risk factors were assessed, such as social deprivation, troubled family history, low educational achievement, irregular employment, history of maltreatment, and violent past. ASD individuals seem to be substantially similar to non-autistic individuals in several studies [19]. On the other hand, comorbid mental diagnoses, such as ADHD, intellectual impairments, psychosis, personality disorder, and other affective disorders, appear to be more prevalent among ASD offenders, but this data may also be associated with a general increased vulnerability of ASD subjects to develop other comorbid psychiatric conditions [62]. Furthermore, some studies suggest that ASD offenders have specific characteristics in the motivation to offend, which comprises social naïveté, social misinterpretations, restricted interests, and peculiar explanations which are part of the core symptoms of this type of neurodevelopmental disorder [19,28,42,64]. In this framework, some authors suggested a correlation between the overrepresentation of autistic features and the kind of criminal activity, such as violent crimes, sexual offending, and stalking [16,57,65,66], while others suggested a deterrent role of the same toward the engagement of criminal activities [47].

From the data reported, however, it is conceivable that subjects with ASD, even when committing crimes, have specific comorbidities, behaviors, types of interaction, and ways of decoding the world, that make these offenders peculiar, highlighting the need for a specific evaluation at each stage of their transit in the CJS. Unfortunately, such specific evaluations and approaches are not frequent and often not possible. In this framework, an analysis of 93 cases from January 2015 to January 2020, aimed to investigate whether ASD subjects’ vulnerabilities were considered at each stage of the CJS, highlighting that in 75% of the cases, reasonable adjustments were not given to diagnosed ASD offenders during the process [22].

Moreover, an appropriate staff during police investigations was offered only in 43% of cases, even though there was a diagnosis of ASD, and 46% of judges and 59% of prosecution barristers reported that they did not have an adequate understanding of autism. Lastly, lawyers were reported to be significantly more worried about the effective participation in court and the self-harm behaviors of their clients [22]. Similarly, another analysis of English case law underlined the lack of training and understanding from advocates, juries, and the judiciary about the specific characteristics of people with ASD, like deficiencies in social awareness and interpersonal skills or poor ability to appreciate non-verbal cues from others. All these clinical features increase the risk of committing and being accused of various types of offending, including sexual ones [23]. This lack of awareness ultimately causes legal omissions and the consequent annulments of the appeal sentences. On the other hand, an analysis of sentencing data from the Australian Bureau of Statistics showed that the lack of knowledge of judges, prosecution barristers, and advocates about the characteristics of ASD lead ASD sex offenders to receive longer sentences compared to sex offenders without ASD [24].

### Limitations

The present paper presents some major limitations. Firstly, our review was not conducted systematically and was limited to English-written articles, although the most recent and significant studies were included. Secondly, some papers were quite old, and several had weak statistical power, due to a limited number of patients analyzed. In addition, many others lacked a standardized and reliable method of diagnosis for autism, with only a few using a clinician’s evaluation. Moreover, most of the research did not have a control group or was heterogeneous, which flawed direct comparisons between groups. Lastly, all these issues, together with the presence of different geographic and socio-cultural environments from where the data was extracted, contributed to a word of caution in the generalization of results.

## 5. Conclusions

In conclusion, although many researchers have focused on understanding criminal conduct among people with ASD, the available studies lack the rigor needed to provide proof for such a broad hypothesis. The link between autistic traits and complex behavior (such as a criminal offense) requires a strong epistemological framework, which should take into account not only psychiatric paradigms but also an adequate and replicable consideration of the social, environmental, and psychological variables that may be involved in criminal conduct. Therefore, the available data do not allow us to establish a link between autistic traits or a diagnosis of ASD and the predisposition to engage in criminal behavior.

## Figures and Tables

**Table 1:** Offending behaviors in ASD subjects.

Authors	Sample	Assessment	Results
Wahlund & Kristiansson, 2006 [25]	Total sample: N = 35 male offenders ASD: N = 8 APD: N = 27	WAIS-R; PCL-R	ASD offenders were less intoxicated at the time of the offense (56% vs. 90%) and had less history of physical abuse (25% vs. 41%). ASD offenders used fewer knives or firearms (11% vs. 71%) and more poisoning, strangulation, and direct force (80% vs. 28%).
Allen D et al., 2008 [11]	Total sample: N = 16 offenders with Asperger Syndrome (mean age: 34.8)	ASDI	All participants had behavioral issues: 69% destructive behaviors, 88% verbal aggression, 75% physical aggression, 69% inappropriate sexual behaviors, 38% over-activity, and 38% illicit drug use. Concurrent psychiatric disorders: 25% schizophrenia, 18.75% ADHD, 12.5% depression, 6.25% anxiety, and 6.25% personality disorders. Predisposing factors: lack of concern for outcome, social naivety, lack of awareness of the outcome, impulsivity, misinterpretation of rules, and overriding obsessions. Precipitating factors: social rejection, bullying, sexual rejection, family conflict, deterioration in mental health, change of domicile and in professional support, and bereavement.
Newman et al., 2008 [26]	N = 37 offenders with Asperger Syndrome		Definite comorbid psychiatric disorder (29.7%): 4 bizarre and antisocial acts, 1 dysmorphophobia, 1 ADHD, and 3 mood disorders. Probable comorbid psychiatric disorder (54%): 1 history of psychiatric admission, 1 obsessive-compulsive disorder and personality disorder, 1 conduct disorder, 1 obsessional neurosis, 4 history of fire-setting, and 3 admission to a forensic hospital. No coexisting psychiatric condition (16.2%).
Lundstrom et al., 2014 [27]	Total sample: N = 3391 ASD: N = 954 ADHD: N = 1366 TD: 214 OCD: N = 157	A-TAC	Subjects with ADHD or TDs were at elevated risk of committing violent crimes and were identified as risk factors for subsequent violent criminality; no such association could be seen for ASD or OCD groups.
Helverschou et al., 2015 [28]	Total sample: N = 48 ASD offenders (M = 41; F = 7)		Late ASD diagnosis (mean age: 25.3 years). No link between the criminal act and ASD, but relationship between the idiosyncratic belief system and obsessions and the crime’s motivations.
Loureiro et al., 2018 [29]	Total sample: N = 211 Male inmates: N = 101 Control group: N = 110	AQ; TriPM; ASRS; BSI	Increased autistic traits in inmates. No correlation between autistic traits and psychopathy. Autistic traits were an independent risk factor for imprisonment.
Slaughter et al., 2019 [30]	Total sample: N = 429 Juvenile ASD offenders: N = 143 Juvenile non-ASD offenders: N = 286		ASD offenders are less likely to commit property offenses. No significant differences in the type of school discipline incidents. ASD subjects are not more likely to commit crimes.
Hofvander et al., 2019 [33]	Total sample: N = 269 Young male ASD offenders: N = 26 Young male non-ASD offenders: N = 243	SCID; ASDI; A-TAC; PCL-R	No significant differences in total PCL-R scores were found. The number of crimes was similar among the groups, but ASD offenders were more likely to report convictions related to sex crimes against child victims, while non-ASD were more involved in drug-related crimes, had more convictions, and more often presented a substance use disorder in comorbidity.
van Buitenen et al., 2021 [34]	Total sample: N = 394 male ASD offenders		Comorbid mental disorders in 78.9% of the sample (39.8% substance use disorder, 14.1% neurodevelopmental disorders, 31.7% schizophrenia spectrum disorders). No direct connection between ASD and the perpetration of violent crimes. Comorbidity, influenceability, and a detrimental social network as indicators of violent conduct.
Yu et al., 2021 [35]	Total sample: N = 4850 ASD: N = 606 ID: N = 1271 PC: N = 2973 DJJ: N = 245 (18 ASD; 87 ID; 140 PC) SLED: N = 333 (20 ASD; 89 ID; 224 PC)		About 3% of young ASD subjects were involved in the DJJ, with few differences between the groups. Adult ASD (3.30%) and ID (7%) were less likely to be involved in the SLED, compared to the PC (7.53%). In both CJSs (DJJ and SLED), the charges more common in the ASD group were crimes against persons, offenses against public order, and crimes against property.
Bowden et al., 2022 [36]	Total sample: N = 149,076 ASD: N = 1197 (M = 945; F = 252) Non ASD: N = 146,863 (M = 75,795; F = 72,084)		ASD had lower rates of being investigated by the police, accused in court, and found guilty. Accused ASD were more likely to be charged with major offenses, violent offenses, offenses against people, and offenses against property.
Blackmore et al., 2022 [37]	Total sample: N = 1570 ASD: N = 1130 (M = 825, F = 305) Non-ASD: N = 440 (M = 317, F = 123)	ADI-R, ADOS-G or ADOS-2	ASD had a lower prevalence of any CJS contact (23% vs. 32%; *p* < 0.01) and committed fewer violent offenses (10% vs. 14%), theft/burglary (5% vs. 9%), and substance offenses (3% vs. 6%). ASD had more co-currying ADHD (20% vs. 11%) and anxiety disorders (51% vs. 43%). Male sex (OR = 3.5, *p*< 0.001) or having ADHD (OR = 1.8, *p* < 0.001) and/or a psychotic disorder (OR = 2.2, *p* = 0.021) resulted to be risk factors for involvement in CJS of ASD patients.
Hofvander et al. [38]	Total sample: N = 831 ASD offenders (M = 708, F = 123)		66.7% of the sample had at least one psychiatric comorbidity. Substance use disorder (26.0%) was the most frequent, followed by schizophrenia (16.1%), ID (15.8%), and ADHD (15.4%). Violent crimes (75.5%) were committed more frequently by ASD subjects, followed by sexual offenses (16.1%), vandalism (13.1%), theft (10.3%), drug-related crimes (7.3%), and others (36.8%).

ASD: autism spectrum disorder; APD: antisocial personality disorder; WAIS-R: Wechsler Adult Intelligence Scale—Revised; PCL-R: Psychopathy Checklist—Revised; ASDI: Asperger Syndrome Diagnostic Interview; ADHD: attention deficit and hyperactivity disorder; TD: Tic disorder; A-TAC: Autism, Tic AD/HD and other Comorbidities Inventory; AQ: Autism Spectrum Quotient; TriPM: Triarchic Psychopathy Measure; BSE: Brief Symptom Inventory; ASRS: Adult ADHD Self-Reported Scale; SCID: Structured Clinical Interview for DSM; ID: intellectual disability; PC: population control group; DJJ: Department of Juvenile Justice; SLED: South Carolina Law Enforcement Division; ADI-R: Autism Diagnostic Interview—Revised; ADOS-G or ADOS-2: Autism Diagnostic Observational Schedule.

**Table 2:** ASD and mass shooting.

Authors	Sample	Results
Allely et al., 2017 [39]	Total sample: 75 mass shooters	6 offenders had a diagnosis of ASD. 22 offenders had significant ASD traits.

ASD: autism spectrum disorder.

**Table 3:** ASD and sex offenses.

Authors	Sample	Assessment	Results
‘t Hart-Kerkhoffs et al., 2009 [40]	Total sample: N = 789 Male juvenile sex offenders: N = 175 (mean age: 14.9 ± 1.4), ASD children: N = 114 (mean age: 14.2 ± 1.9) HCs: N = 500 (mean age: 14.0 ± 1.4)	CSBQ	Sex offenders had higher CBQ scores than HCs but lower than the ASD group.
Ewoud Baarsma et al., 2016 [41]	Total sample: N = 96 Male sex offenders: N = 44 (mean age: 24.7 ± 1.5) HCs: N = 52 (mean age: 24.3 ± 0.7)	SOJ25II; CSBQ; ASBQ	No differences in sexual development milestones. Stability of autistic symptoms over time. No relationship between sexuality and either sexual development or ASD symptoms.
Payne et al., 2019 [42]	Total sample: N = 9 male ASD sexual offenders (mean age: 29.56 ± 8.68)	Semi-structured interviews	Five main reasons for the crime: social difficulties, misunderstanding, sex and relationships, inadequate control, and disequilibrium.
Rutten et al., 2022 [43]	Total sample: N = 188 males ASD: N = 69 ADHD: N = 90 ASD + ADHD: N = 29	76-items checklist	Significantly higher rates of sex offenses in ASD compared to the other groups. No differences in violent offending rates between groups.

CSBQ: Children’s Social Behavior Questionnaire; SOJ25II: Sex under the Age of 25-II questionnaire; ASBQ: Adult Social Behavior Questionnaire.

**Table 4:** ASD and cyber crimes.

Authors	Sample	Assessment	Results
Seigfried-Spellar et al., 2014 [45]	Total sample: N = 296 university students	AQ; CCI-R	AQ score: non-hackers > hakers; cyberbullies, identity thieves, virus writers > non-deviant counterparts. Participants who self-reported engaging in all four types of cyber-deviancies had higher scores in the total AQ as well as more problems with imagination, social skills, and communication.
Payne et al., 2019 [46]	Total sample: N = 290 (M = 194; F = 94; mean age:24.24 ± 9.25) Self-reported ASD: N = 23	AQ; RAPM; IToSK; ISEL-12; BADS: CDCQ	Higher AQ scores in subjects who acted ou a cybercrime. An increased risk of committing cybercrime with higher autistic-like traits, but a lower risk of committing cybercrime with a diagnosis of ASD. Having advanced digital skills mediated 40% of the association between cybercrime and autistic traits.
Payne et al., 2020 [47]	Total sample: N = 36 CDD: N = 29 (M = 16; F = 13; mean age: 28.59 ± 9.16) CDO: N = 7 (M = 7; mean age: 18.29 ± 3.30)	RAPM; AQ-50; BADS	Higher IQ in the CDD group. Lower mean AQ-50 score in the CDO compared to CDD. Autistic traits are related to reasons for declining to commit cyber-dependent offenses.
Lim et al., 2023 [49]	Total sample: N = 302, ASD: N = 25	AQ-12; 10-item basic digital skills questionnaire, 10-item advanced digital skills questionnaire; A-ToM-Q; AVT; CDCQ; CCI-R	ASD subjects were more engaged in criminal activity. Gender, A-ToM-Q, AQ-12, and AVT scores or basic and advanced digital skills did not present significant correlations with the endorsement of cybercrime. Measuring cybercriminal activity with the CDCQ, verbal comprehension and advanced digital skills were associated with more cyber-dependent crime.

AQ: autism quotient; CCI-R: Computer Crime Index—Revised; RAPM: Ravens Advanced Progressive Matrices Set I; IToSK: informal test of social know-how; ISEL-12: Interpersonal Support Evaluation List-12: BADS: Basic and Advanced Digital Skills; CDCQ: Cyber-Dependent Crime Questionnaire; CDD: cyber-dependent decliners; CDO: cyber-dependent offenders; A-ToM-Q: Adult Theory of Mind—Quick; AVT: Advanced Vocabulary Test I–V-4; CCI-R: Computer Crime Index—Revised.

## Data Availability

No new data were created or analyzed in this study.
